# The role of CSE1L expression in cervical lymph node metastasis of larynx tumors

**DOI:** 10.1016/j.bjorl.2019.06.010

**Published:** 2019-07-24

**Authors:** Tuncay Tunccan, Sertac Duzer, Gulay Dilek, Ulvi Murat Yuksel, Hasan Cetiner, Caner Kılıc, Ayca Ant, Arzu Betul Duran

**Affiliations:** a Oncology Training and Research Hospital, Department of Ear Nose and Throat, Ankara, Turkey; b Elazig Training and Research Hospital, Department of Ear Nose and Throat, Elazig, Turkey; c Oncology Training and Research Hospital, Department of Pathology, Ankara, Turkey; d Oncology Training and Research Hospital, Department of Surgery, Ankara, Turkey

**Keywords:** T3–T4 glottic cancer, CSE1L, Lymph node, Metastasis

## Abstract

**Introduction:**

According to international reports, 30–40% of all head and neck cancers are larynx cancers, comprising 1–2.5% of all cancer types. Cervical nodal involvement has been reported to be 40% and 65% in T3 and T4 cases, respectively. Five-year survival in patients with cervical lymph node metastasis has been demonstrated to be 50% lower compared to patients with no metastasis. Chromosome segregation like 1 protein; is a DNA fragment isolated by Brinkmann et al. in 1995 that corresponds to yeast chromosome segregation protein. Studies on the effect of chromosome segregation like 1 protein expression in head and neck tumors are rare and it has been shown that nuclear chromosome segregation like 1 protein is over-expressed in these studies where gastrointestinal and breast tumors over-expressed cytoplasmic chromosome segregation like 1 protein.

**Objective:**

Chromosome segregation like 1 protein may regulate the proliferation and metastasis of T3–T4 glottic larynx cancer. The aim of this study is to show the relationship between chromosome segregation like 1 protein expression and cervical lymph node metastasis of T3–T4 glottic larynx cancer.

**Methods:**

A total of 57 male patients who were operated for T3–T4 glottic cancer in a tertiary referral hospital was included in this study. There were 28 patients with cervical lymph node metastasis and 29 patients without lymph node metastasis. Immunohistochemistry was carried out on formalin-fixed, paraffin-embedded archival glottic larynx tumour tissue. According to the percentage of immunoreactive cells, chromosome segregation like 1 protein status was analyzed.

**Results:**

Among the patients, who had no cervical lymph node metastasis, 15 patients showed weak nuclear staining, 12 patients showed moderate nuclear staining and only 2 patients showed high nuclear staining for chromosome segregation like 1 protein. Among the patients who had cervical lymph node metastasis, 18 patients showed high nuclear staining, 9 patients showed moderate staining and only one patient showed weak staining for chromosome segregation like 1 protein. None of the metastatic patients showed cytoplasmic staining and only one patient in the non-metastatic group showed cytoplasmic staining for chromosome segregation like 1 protein. There was a positive correlation between nuclear chromosome segregation like 1 protein expression and cervical lymph node metastasis (*r* = 0,668) and it was statistically significant (*p* < 0,001).

**Conclusion:**

Chromosome segregation like 1 protein expression is correlated with lymph node metastasis in T3–T4 glottic cancers. This may change the approach to cervical node treatment in patients with glottic cancers in future.

## Introduction

According to international reports, 30–40% of all head and neck cancers are larynx cancers, comprising 1–2.5% of all cancer types.^1^ Pathology reveals squamous cell cancer in 95–98% of them. The disease is more prevalent in the male sex and it is incidence increases in the fifth and seventh decades.^2^ The disease occurs most frequently in the glottic region, less in supraglottic region and subglottic region respectively. The rate of clinical presence of cervical metastasis has been reported to be 51% and the rate of cervical nodal metastasis increases with the advancement of the stages of the primary tumor. Cervical nodal metastasis has been reported to be 40% and 65% in T3 and T4 cases, respectively.^3^ The Paraglottic Space (PGS) and epiglottis invasion increase cervical nodal metastasis rate; impaired or fixed vocal cord mobility are the risk factors for PGS invasion and cervical lymph node metastasis.^4^ In recent years, with the improvement in molecular biology there have been different agents which are in both clinical and experimental used to determine lymph node metastasis. Chromosome Segregation Like 1 protein (CSE1L) is one of them.

Chromosome Segregation Like 1 protein (CSE1L) is a DNA fragment isolated by Brinkmann et al. in 1995 that corresponds to yeast chromosome segregation protein. There are two forms of CSE1L: cytoplasmic and nuclear CSE1L.^5^ It is highly expressed in tissues that have a high mitotic index such as human tumor cells, fetus liver and testes. Apopitosis, cell survival, chromosome installation and nucleocytoplasmic transport are known functions of CSE1L. It also plays a role in the regulation of P53 tumor supressor gene and increases tumor invasion by enhancing Matrix Metalloproteinase-2 (MMT-2) secretion in tumoral tissue. For this reason, in some cancer types CSE1L is thought to be related to cancer progression and metastasis.^6^ Studies on the effect of CSE1L expression in head and neck tumors are rare and it is shown that nuclear CSE1L is over-expressed in these studies where gastrointestinal and breast tumors over-expressed cytoplasmic CSE1L.^7^

Our aim was to find out whether there is a correlation between CSE1L expression and regional lymph node metastasis in advanced stage larynx tumors.

## Methods

Patients who were operated because of T3 and T4 glottic cancer between January 2008 and July 2016 in Ear-Nose and Throat Diseases Department of Ankara Oncology Training and Research Hospital were evaluated. The study protocol was approved by the Ethics Committee of Ankara Oncology Training and Research Hospital (Project no 2017-6/97), Ankara, Turkey. Fifty seven male patients were included in the present study. They received either total or supracricoid partial laryngectomy. Bilateral level II, III, IV lateral neck dissection was applied on all the patients. The patients were staged according to American Joint Cancer Commitee (AJCC) 8th edition. Paraglottic extent was invaded in all of the T3 patients. Preepiglottic region invaded patients were excluded. Age, T stage, grade of the patients, cervical lymph node status and the operation type were recorded.

Immunohistochemistry was carried out on formalin-fixed, paraffin wax covered archival tissue. CSE1L staining was performed on 5 μm (micro-meter) thick slides using the GeneTex CSE1L antibody rabbit polyclonal antibody (1:100) using a Ventana Ultraview DAB detection kit in a Ventana BenchMark XT processor (Ventana, Tucson, AZ). Antigen retrieval was a standard automated process on Ventana BenchMark XT at 37 °C for 16 min. The sites of peroxidase activity were visualized using diaminobenzidine (3,3′-diaminobenzidine tetrahydrochloride) as the substrate and counterstained using Mayer’s hematoxylin.

### Immunohistochemical scoring system

**Figure 1:**
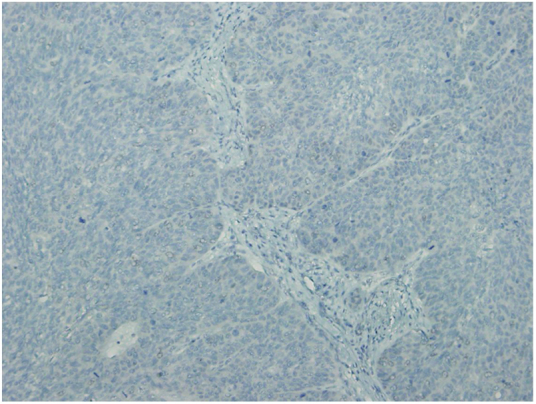
Non-metastatic squamous cell carcinoma; negative CSE1L staining.

**Figure 2:**
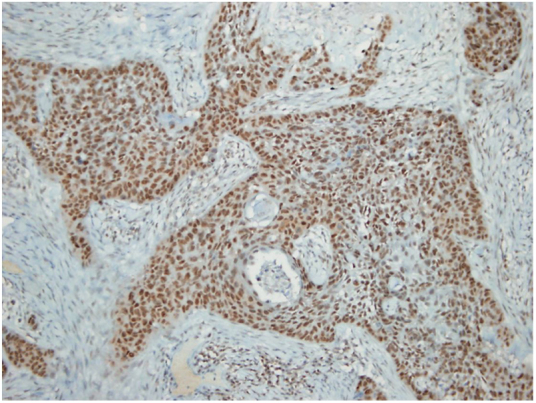
Metastatic squamous cell carcinoma; positive nuclear CSE1L staining.

Semiquantitative scoring system was used to analyze immunohistochemical staining; the percentage of immunoreactive cells was combined with the estimated staining intensity. According to these two combined parameters the tumor was scored in terms of nuclear or cytoplasmic staining. The staining intensity score varied from 1 to 3. For example score 1 showed weak staining, score 2 showed moderate staining and score 3 showed strong staining. A calculation was made to find out the total possible intensity score 300 as: (1 × percentage of weak stained tumor cells) + (2 × percentage of moderate stained tumor cells) + (3 × percentage of strong stained tumor cells). Finally, the staining results were categorized in low-CSE1L (CSE1L staining 1+), modarate-CSE1L (CSE1L staining 2+) and high-CSE1L (CSE1L staining 3+) subgroups. The representative immunohistochemical images of nuclear CSE1L staining are shown in Figure 1, Figure 2.

### Statistical analyses

Statistical analyses were performed by using the SPSS software version 21. Variables were investigated by using visual (histogram, propability plots) and analytical methods (Kolmogorov–Smirnov/Shapiro–Wilk’s tests) to determine whether they are normal or not. While investigating the associations between non-normally distributed and ordinal variables, the correlation coefficients and their significance were calculated by using the Spearman test. A 5% Type 1 error level was used to infer statistical significance.

## Results

57 male patients squamous cell carcinoma of the glottis region were included in the study; the mean age was 59.3 years. Twenty seven of the patients had T3 and the rest of them had T4 tumor stage. Cervical lymph node involvement was present in 28 (49%) patients and the median number of metastatic lymph node was 2.6 (range 1–8). The tumor was low grade (Grade 1) in 25 patients and medium grade (Grade 2) in 28 patients; the grade was unknown in 4 patients. None of the patients had high grade (Grade 3) tumor. In the patients who had no cervical lymph node metastasis, 15 patients showed weak nuclear staining, 12 patients showed moderate nuclear staining and only 2 patients showed high nuclear staining for CSE1L. Among the patients who had cervical lymph node metastasis, 18 patients showed high nuclear staining, 9 patients showed moderate staining and only one patient showed weak staining for CSE1L. None of the metastatic patients showed cytoplasmic staining and only one patient in the non-metastatic group showed cytoplasmic staining for CSE1L. There was a positive correlation between nuclear CSE1L expression and cervical lymph node metastasis (*r* = 0.668) and it was statistically significant (*p* < 0.001). The number of metastatic cervical lymph nodes was not correlated with CSE1L staining intensity. In addition, the correlation between CSE1L staining and other parameters (tumor size, grade and extension of neck dissection) was also investigated (Table 1), however there was not any statistically significance.

**Table 1:** Investigated parameters and their correlations with CSE1L.

	T stage (n = 57)	Grade (n = 53)	Metastasis (n = 57)	Neck dissection type (n = 57)
	(Coeff) *p*	(Coeff) *p*	(Coeff) *p*	(Coeff) *p*
CSE1L expression	(−0.091) 0.502	(0.062) 0.661	(0.668) <0.001	(−0.136) 0.312

Correlation is significant, *p* < 0.001 (2 tailed).

## Discussion

Cervical nodal metastasis is a common cause of poor survival in larynx cancer, like all the head and neck tumors. Five year survival rates are diminished down to 30% in these cases. Cervical nodal metastasis rates are reported as 15–65% in literature.^8^ In advanced stage larynx and hypopharynx tumors, cervical nodal metastasis rates are related to the invasion of different anatomical sites. For instance, the rate of cervical nodal metastasis is almost 100% if the sinus priformis is invaded, and it is 70% if the tumor invaded of full thickness thyroid cartilage where it is about 40–50% in case of the invasion of other anatomical sites. In our study the rate of cervical nodal metastasis in cases of the paraglottic region invasion in advanced stage patients was compatible with the literature.

CSE1L is localized on chromosome 20q13 and this gene regulates multiple cellular mechanisms, including the mitotic spindle checkpoint, basically proliferation and apoptosis which are two opposing processes.^9^ CSE1L is synthesized in response to cellular stress and, in non-neoplastic cells, and is usually localized to the nucleus. CSE1L also plays a role in the nuclear-cytoplasmic reshuffling of importin-α,^10^ in microtubule-associated cellular proliferation, in the migration and invasion of cancer cells^7^ and in the regulation of suppressor genes. Furthermore, low CSE1L expression resulted in inhibition of the metastasis of tumor cells in animal models; for example, it inhibited the metastasis of B-16 and F10 melanoma cells in mice.^11^ There are some recent studies sustaining the effect of CSE1L on cancer proliferation, too.^12^

CSE1L expression is required for both cell division and true sequence of the chromosomes that are responsible for genomic stability. For this reason, abnormal expression of CSE1L may affect genomic stability favoring the cancer progression. In our study, we tried to find out the relationship between CSE1L expression and cervical nodal metastasis in larynx tumors. There are limited studies concerning CSE1L expression in head and neck tumors and they are experimental in vitro studies in which progression and invasion were evaluated with multiple genes.^13^ The present study is the first in vivo study on the topic that may be considered as an attempt to build an awareness on head and neck tumors.

CSE1L affects tumor behavior by changing the function of various genes. In the study of Cheng et al.;^14^ CSE1L increased the expression of MutS Homolog 6 (MSH6) on osteosarcoma patients and it is found to be a poor prognostic factor for causing cancer progression. On the other hand, Liao et al.^15^ showed that CSE1L over-expression did not enhance cancer proliferation but reducted CSE1L inhibited metastasis. Li et al.^16^ revealed that MicroRNA-137 (miR-137) is the target gene of CSE1L in ovary cancer and plays a role in miR-137 related tumor supression. Lorenzato et al.^17^ found out that CSE1L regulates RASSF1C expression and in this way inhibits apopitosis in ovary cancer cells. Jiang et al.^18^ showed the importance of cytoplasmic CSE1L expression on malignant transformation of Barrett esophagus.

Related to head and neck cancers in one of the rare studies on the topic, Kim et al.^12^ claimed that CSE1L was an ineffective prognostic parameter in nasopharynx cancer patients. However, Fang et al.^13^ reported that CSE1L plays a role in apopitosis, invasion and metastasis of nasopharynx cancer. According to Soldini et al.^19^ CSE1L is a useful parameter to distinguish Burkitt lymphoma and diffuse large B-cell lymphoma, nonetheless treatment can be planned in intermediate forms according to CSE1L expression level.

We thought it would be a suitable work to search the clinical value of CSE1L in T3–T4 glottic larynx cancers and investigated its correlation with cervical lymph node metastasis. Glottic region cancers metastasize to regional cervical lymph nodes more rarely than other larynx tumors and glottic region cancers are more common than other larynx tumors. For this reason, we preferred to investigate CSE1L expression in glottic cancers. We studied both nuclear and cytoplasmic expression of CSE1L by immunohistochemistry on primary tumor and tried to find any correlation with this protein and clinicopathological characteristics such as T phase, grade, extension of cervical dissection (unilateral and bilateral) as well as cervical lymph node metastasis. In this study, it is found that nuclear CSE1L is correlated with cervical lymph node metastasis (*p* < 0.001), but it was not correlated with grade, tumor size and the extension of neck dissection. Yuksel et al.^20^ found the relationship between cytoplasmic CSE1L overexpression and axillary lymph node metastasis in breast cancer, however, they could not find any relationship between nuclear CSE1L and axillary lymph node metastasis. There was only one patient who presented cytoplasmic CSE1L staining which is in the non-metastatic group in our study. This difference can be explained by the different type and the site of the tumor may be responsible of this result.

Except for selected T3 tumors, unilateral or bilateral level I‒IV neck dissection is the part of the standard treatment procedure for both T3 and T4 tumors at the present day. Neck dissection may have result in complications such as bleeding, hematoma, epidermolysis and various iatrogenic nerve injuries. In some cases, neck dissection may be an unnecessary surgery which extends the operation duration and as well as hospital stays. If future prospective randomized controlled studies support the findings of this study, surgeons can elecy to avoid neck dissection in selected patients.

## Conclusion

CSE1L over expression is correlated with cervical lymph node metastasis in T3–T4 glottic cancers and this may change the approach to neck treatment of larynx cancers.

## Conflicts of interest

The authors declare no conflicts of interest.
